# Operationalising neuroinclusive work systems: A multi-method framework for organisational research

**DOI:** 10.1016/j.mex.2026.104098

**Published:** 2026-08-14

**Authors:** Beth Tootell, Vasudha Rao

**Affiliations:** School of Management and Marketing, Massey University, Palmerston North, New Zealand

**Keywords:** Neurodiversity, Human resource management, Work design, Job demands-resources, Organisational inclusion

## Abstract

Research on neurodiversity in employment has expanded rapidly, but organisational studies remain fragmented across employee experiences, accommodation practices and recruitment initiatives. The absence of a standardised method for operationalising neuroinclusive work systems limits comparison, replication and cumulative theory development. This article addresses that gap by presenting a replicable four-phase framework integrating conceptual synthesis, longitudinal panel modelling, organisational multilevel analysis and experimental recruitment simulation.This article presents a multi-method research framework for operationalising neuroinclusive work systems in organisational research. The framework integrates four complementary methodological components: conceptual synthesis, longitudinal panel modelling, organisational multilevel analysis, and experimental recruitment simulation. Drawing on HRM systems theory and the Job Demands–Resources (JD-R) framework, the method enables researchers to examine how organisational systems generate demand-resource environments that influence employee wellbeing, job satisfaction, retention, and earnings outcomes. The framework is designed for replication across publicly available panel datasets, linked employer–employee datasets, and controlled recruitment experiments. This article provides procedural guidance for implementing the framework and demonstrates how triangulation across methods strengthens inference regarding work accessibility.

## Highlights

• Integrates conceptual, longitudinal, multilevel, and experimental methods.
• Operationalises neuroinclusive work systems using established datasets.
• Provides a replicable framework for examining work accessibility.

## Graphical abstract

**Figure:**
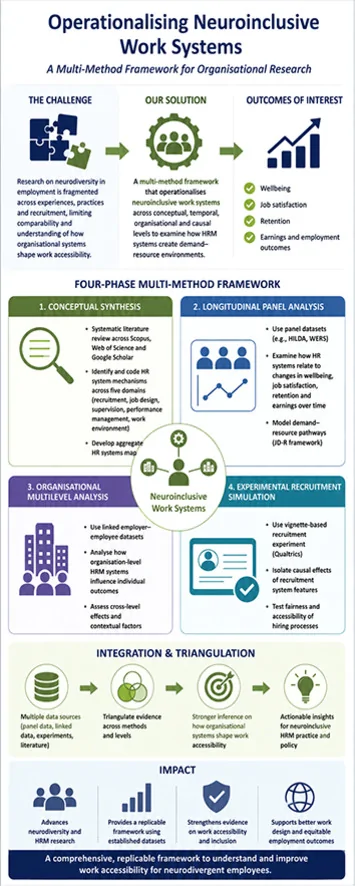

**Specifications table**

**Table:** 

Subject area	Economics and Finance
**More specific subject area**	
Human Resource Management	
**Name of your method**	Multi-method neuroinclusive work systems framework [1]
**Name and reference of original method**	Job Demands–Resources model [2]; HRM systems theory [3]
**Resource availability**	Publicly available datasets (HILDA, WERS), Qualtrics experimental platform, R/Stata/SPSS syntax

## Background

Organisational research on neurodiversity has expanded considerably in recent years, but methodological approaches remain fragmented. Existing studies have primarily focused on employee experiences, employer perceptions, or accommodation practices, often relying on single-method cross-sectional designs. While these studies have generated important insights, they provide limited understanding of how organisational systems shape the accessibility of work itself.

A growing body of conceptual work suggests that work accessibility is not solely determined by individual cognitive characteristics but is structured through organisational systems, including recruitment processes, job design, communication practices, and performance management systems. These systems create patterns of job demands and job resources that influence how work is experienced across different cognitive profiles.

Despite growing scholarly interest in neurodiversity and employment, existing organisational research has largely focused on isolated aspects of workplace experience, including disclosure, accommodations, recruitment practices, and employee perceptions. Although these studies have generated important insights, they have not produced a consistent methodological approach for operationalising neuroinclusive work systems or examining how multiple HR practices interact to influence workplace accessibility. Consequently, findings remain difficult to compare across studies, limiting opportunities for cumulative theory development and replication. The present framework addresses this methodological gap by integrating complementary methodological approaches within a single operational model that can be applied consistently across organisational research.

The proposed framework is grounded in the neurodiversity paradigm, which shifts attention from individual deficit towards the interaction between cognitive characteristics and social and organisational environments [4]. Emerging organisational research similarly demonstrates that employment outcomes for neurodivergent people are shaped by recruitment systems, job design, supervisory practices and the broader organisational context [[5], [6], [7]]. This perspective aligns with HRM systems theory and the Job Demands–Resources framework, both of which recognise that organisational practices shape employee experiences, wellbeing and performance through the configuration of workplace demands and resources.

The companion conceptual article associated with this method paper develops an integrative framework explaining how clinical, educational, and organisational definitions of neurodiversity are translated into HR systems. The present article operationalises that framework by providing a multi-method design for empirical testing. The proposed framework distinguishes neurodivergence from acquired health conditions that may also affect workplace functioning. Neurodivergence refers to naturally occurring differences in cognitive processing that are generally present across the lifespan, whereas conditions such as cancer, diabetes, or physical injury may produce functional limitations through different biological mechanisms. Although both groups may encounter workplace barriers and require organisational support, the mechanisms underpinning these experiences differ conceptually. Accordingly, this framework focuses specifically on organisational accessibility for employees with neurodivergent cognitive profiles rather than on disability or health conditions more broadly. Accordingly, this framework focuses specifically on organisational accessibility for employees with neurodivergent cognitive profiles rather than on disability or health conditions more broadly. Importantly, the framework does not assess or diagnose individual employees. Its object of assessment is the accessibility of organisational systems and work environments for people with differing cognitive profiles.

The methodological challenge in this field is that no single design can adequately capture the system-level, individual-level, and causal processes involved. Longitudinal panel data enable analysis of employment and wellbeing outcomes over time, but provide limited organisational process detail. Linked employer–employee datasets provide organisational-level HR system data, but less temporal depth. Experimental methods isolate recruitment system effects but sacrifice external realism.

No single methodological approach can simultaneously establish conceptual clarity, examine longitudinal change, capture organisational context, and identify causal mechanisms underlying neuroinclusive work systems. Accordingly, the proposed framework integrates four complementary methodological approaches that collectively strengthen methodological triangulation, improve robustness, and provide a more comprehensive understanding of how organisational systems shape workplace accessibility.

This article addresses these limitations by integrating four complementary methodological approaches into a unified research framework. The method enables triangulation across conceptual, longitudinal, organisational, and experimental evidence, improving robustness and replicability in neurodiversity and HRM research.

## Method details

This method operationalises neuroinclusive work systems through a four-phase multi-method design integrating conceptual synthesis, longitudinal panel analysis, organisational multilevel modelling, and experimental recruitment simulation. The design enables examination of work accessibility across multiple levels of analysis and supports stronger inference through triangulation across complementary methods.

The four phases are designed sequentially but may also be implemented independently depending on data availability and research context.

### Phase 1: conceptual synthesis protocol

The first phase identifies and classifies organisational HR system mechanisms associated with work accessibility for neurodivergent employees. The purpose is to develop a structured coding framework for identifying how HR practices shape demand-resource configurations. A structured literature search should be conducted across Scopus, Web of Science, and Google Scholar following the Preferred Reporting Items for Systematic Reviews and Meta-Analyses (PRISMA) guidelines to improve transparency and replicability of the search process [8].

The literature search should be undertaken across Scopus, Web of Science, PsycINFO, and Google Scholar using combinations of keywords including neurodivers, autism, ADHD, dyslexia, and dyspraxia combined with employment, workplace, organisation, human resource management, and HRM, (neurodivers* OR neurodiverg* OR autis* OR ADHD OR “attention deficit hyperactivity disorder” OR dyslex* OR dyspraxi*) AND (employ* OR workplace* OR organisation* OR organization* OR “human resource management” OR HRM OR recruitment OR “job design” OR accommodation*). Searches should be limited to peer-reviewed English-language publications published between 2010 and 2025. Following duplicate removal, titles and abstracts should be independently screened by two reviewers before potentially eligible studies undergo full-text review. Disagreements regarding study eligibility should be resolved through discussion until consensus is achieved, with reference list screening undertaken to identify any additional eligible studies.

Studies will be included where they examine neurodiversity or neurodivergence within organisational or workplace settings and provide empirical or conceptual insight into human resource management practices or work design. To ensure relevance to the organisational focus of the framework, included studies must address employment-related systems, practices, or employee experiences and be published in peer-reviewed outlets in the English language. Both empirical and conceptual contributions will be retained where they contribute to understanding how organisational systems shape work accessibility. Consistent with PRISMA 2020 recommendations, the study selection process should be documented using a PRISMA flow diagram reporting the number of records identified, screened, excluded, assessed for eligibility, and included in the final conceptual synthesis. Reporting these stages improves transparency and facilitates replication of the review process.

Studies will be excluded where their primary focus is limited to educational settings or clinical treatment contexts without organisational application. Opinion pieces, practitioner commentaries, and non-peer-reviewed publications will also be excluded unless they provide substantive conceptual development directly relevant to organisational systems.

Following article selection, each study will be coded using a structured framework designed to identify recurring organisational mechanisms associated with neuroinclusive work systems. Coding will focus on five core HR system domains. The first domain, recruitment systems, will capture the extent to which hiring processes are structured, the types of assessment used, and the communication formats required of applicants. The second domain, job design, will focus on levels of autonomy, predictability, and flexibility embedded within work roles, reflecting broader work design theory emphasising the relational and proactive structuring of work environments [9]. The third domain, supervision practices, will examine the provision of feedback, managerial support, and role clarity. The fourth domain, performance management systems, will assess the clarity of performance criteria and the extent to which behavioural expectations are formalised or implicit. The final domain, work environment characteristics, will capture sensory demands, interruption frequency, and communication norms embedded in everyday work practices.

This coding structure enables systematic identification of how HR systems shape the demand-resource architecture of work environments and provides the basis for operationalisation in subsequent phases of the research programme.

To improve analytical consistency, coding will be conducted independently by two researchers. In the first stage, each coder will independently review and classify studies against the five HR system domains. Following independent coding, the coders will compare their classifications to assess agreement and identify discrepancies. Any coding disagreements will be resolved through discussion and re-examination of the source material until consensus is reached. Final coding outputs will then be synthesised into an aggregate HR systems map identifying recurring organisational practices associated with work accessibility.

Intercoder reliability will be assessed using Cohen's kappa to evaluate coding consistency across the five domains. Consistent with established methodological recommendations, a threshold of κ ≥ .70 will be used as the minimum acceptable level of agreement. Where agreement falls below this threshold, coding categories will be reviewed and refined before re-analysis.

The final output of Phase 1 will be a structured neuroinclusive HR systems framework identifying the organisational practices most consistently associated with accessible work environments. This framework will provide the operational foundation for variable selection and hypothesis testing in the longitudinal, multilevel, and experimental phases of the broader research design.

### Phase 2: longitudinal panel operationalisation (HILDA)

The second phase examines how work-system demand-resource configurations influence employee outcomes over time. This phase is designed to test whether variations in job demands and job resources are associated with changes in employee wellbeing, job satisfaction, employment retention, and wage outcomes, and whether these relationships differ for employees with health conditions or disabilities.

This phase uses the Household, Income and Labour Dynamics in Australia Survey (HILDA), a nationally representative longitudinal household panel survey designed to examine employment, income, and wellbeing trajectories over time [10]. The longitudinal structure of the dataset enables examination of within-person change over time and supports stronger temporal inference than cross-sectional approaches.

The analytical sample will include working-age employees between 18 and 65 years of age who are employed at baseline and observed across a minimum of two consecutive survey waves. Participants who are retired or unemployed at baseline will be excluded from analysis. Based on standard HILDA participation patterns, the expected analytical sample is estimated to range between 8000 and 12,000 respondents across multiple waves.

Job demands will be operationalised using established HILDA measures capturing workload intensity, time pressure, and emotional strain. Job resources will be operationalised through measures of autonomy, supervisor support, and job flexibility. These variables will be standardised and combined into composite demand and resource indices to improve comparability across constructs.

The primary outcome variables include employee wellbeing, job satisfaction, retention in employment, and wage progression. Wellbeing and job satisfaction will be treated as repeated continuous measures, while retention will be operationalised through continued employment status across subsequent waves. Wage outcomes will be measured using logged earnings to improve distributional normality.

Health or disability status will be included as a moderating variable to assess whether the effects of work-system demand-resource configurations differ for employees with health conditions or disabilities. This variable will be coded dichotomously, distinguishing employees who report a health condition or disability from those who do not. It is acknowledged that HILDA does not specifically identify neurodivergent employees. HILDA does not identify neurodivergent employees, and health or disability status is therefore not treated as a proxy for neurodivergence. Instead, this phase provides a broader test of whether demand–resource relationships differ for employees reporting long-term health conditions or disabilities. Findings from this phase cannot be interpreted as estimates of outcomes specific to neurodivergent employees.

The analytical strategy will use fixed-effects longitudinal regression models to estimate within-person changes over time while controlling for unobserved time-invariant individual characteristics. This approach reduces bias associated with stable individual differences and is appropriate for examining how changing work conditions relate to changing employee outcomes.

$$Y_{\text{it}}=\beta_{0}+\beta_{1}D_{\text{it}}+\beta_{2}R_{\text{it}}+\beta_{3}{\left(\text{DR}\right)}_{\text{it}}+\alpha_{i}+\epsilon_{\text{it}}$$

In this model, employee outcomes are modelled as a function of job demands, job resources, and their interaction over time. The estimation procedure involves constructing composite demand and resource indices, standardising all predictor variables, estimating fixed-effects models, and testing interaction effects to identify whether resource-rich work environments buffer the effects of job demands.

To strengthen robustness, sensitivity analyses will be conducted using alternative model specifications, including random-effects models, lagged dependent variable models, and clustered standard errors.

### Phase 3: organisational multilevel modelling (WERS)

The third phase examines how organisational HR practices shape employee experiences of work accessibility by linking organisational-level HR systems to employee-level demand-resource experiences and outcomes.

This phase uses the Workplace Employment Relations Study (WERS), which links employer-level HR system information with employee-level workplace experiences. The dataset enables examination of organisational systems and employee outcomes simultaneously, making it particularly suitable for multilevel analysis.

The dataset has a nested structure, with employees clustered within organisations. The expected analytical sample includes >300 organisations and approximately 20,000 employees.

Phase 3 uses WERS 2011, the most recent completed wave of the Workplace Employment Relations Study. WERS 2011 obtained productive management interviews from 2680 workplaces and 21,981 employee questionnaires, with employee responses received from 1923 workplaces; the final linked analytical sample will be smaller following matching and missing-data exclusions. Its linked employer–employee structure remains valuable for examining relationships between workplace-level HR practices and employee experiences. Nevertheless, the age of the data is a substantive limitation. WERS cannot represent contemporary neurodiversity awareness, post-pandemic hybrid working or more recent neuroinclusive practices. This phase is therefore used to examine historically situated structural relationships between HR practices, job demands, job resources and employee outcomes—not to estimate current neurodivergence prevalence or contemporary organisational practice. Findings will be explicitly bounded to the 2011 British workplace context and will require replication using more recent linked data.

Organisational-level HR system variables will include structured recruitment processes, flexible working practices, supervisor training systems, formal performance management systems, and employee support mechanisms. These measures are derived from the employer survey component of WERS.

Employee-level variables will include job demands, job resources, job satisfaction, and wellbeing. These measures are derived from the employee survey component and will capture individual experiences of the work environment.

Organisational HR practices will be operationalised at the organisational level using management survey responses. Employee experiences will remain at the individual level and be nested within organisational units. To assess the appropriateness of aggregation, standard multilevel diagnostics will be conducted, including intraclass correlation coefficients (ICC1 and ICC2) and within-group agreement estimates (rwg).

Multilevel modelling will be used to account for the nested structure of the data and examine both within-organisation and between-organisation effects.

The employee-level model is specified as:

$$Y_{\text{ij}}=\beta_{0j}+\beta_{1j}X_{\text{ij}}+r_{\text{ij}}$$

The organisational-level model is specified as:

$$\beta_{0j}=\gamma_{00}+\gamma_{01}HR_{j}+u_{0j}$$

The analytical procedure begins by estimating a null model to assess between-organisation variance. This is followed by calculation of intraclass correlations, inclusion of employee-level predictors, addition of organisational HR system variables, and testing of cross-level interaction effects. This procedure enables identification of organisational practices associated with more accessible work environments.

### Phase 4: experimental recruitment simulation

The fourth phase isolates the causal effects of recruitment system design on hiring evaluations by experimentally manipulating recruitment structure and candidate communication style.

Participants will consist of managers and HR professionals recruited through professional networks, including LinkedIn, professional HR associations, and industry contacts. These participant groups were selected because they are directly involved in employment decision-making and provide greater ecological validity than student samples.

A minimum sample of 240 participants will be targeted based on an a priori power analysis designed to detect medium-sized effects with 80% power at an alpha level of .05.

The study uses a 2 × 2 factorial design manipulating two independent variables, consistent with recommendations for designing organisational experiments that maximise both internal validity and generalisability [11].

Recruitment structure is manipulated at two levels: structured and unstructured. Communication style is manipulated at two levels: polished and less polished. This produces four experimental conditions: structured-polished, structured-less polished, unstructured-polished, and unstructured-less polished

Participants will be randomly assigned to one of the four experimental conditions. Each participant will review a standardised applicant package consisting of a curriculum vitae, interview transcript, and work sample. The content of these materials will remain constant across conditions, with only the manipulation variables changing.

After reviewing the materials, participants will complete candidate evaluations assessing hiring recommendation, perceived competence, perceived organisational fit, and decision confidence.

Manipulation checks will assess whether participants perceived the intended differences in recruitment structure and communication fluency. These checks will be included immediately after the evaluation task to assess manipulation strength.

The primary analytical approach will use factorial ANOVA to examine main effects and interaction effects across the four experimental conditions. Where significant interaction effects are identified, simple effects tests and post-hoc comparisons will be conducted to identify specific condition differences.

### Integration procedure

The four methodological phases are integrated through a sequential triangulation design. Each phase contributes a different form of evidence to the broader neuroinclusive work systems framework. Phase 1 establishes the conceptual boundaries of neuroinclusive HR systems and identifies recurring organisational mechanisms. Phase 2 examines longitudinal employee outcomes associated with demand-resource configurations. Phase 3 examines organisational-level HR system effects using linked employer-employee data. Phase 4 isolates causal mechanisms within recruitment systems under controlled experimental conditions. Triangulation occurs through convergence assessment across methods. Where findings converge across conceptual, longitudinal, multilevel, and experimental evidence, confidence in the robustness of the framework is strengthened. Where findings diverge, these differences help identify contextual contingencies and boundary conditions.

This integration strategy improves internal and external validity by reducing reliance on any single data source or methodological approach.

## Method validation

Validation of the framework occurs through four complementary mechanisms. First, construct validity is strengthened through the use of established operational measures in both HILDA and WERS, ensuring that demand-resource constructs and HR system variables are grounded in validated instruments. Second, triangulation validity is established by testing the same conceptual framework across multiple methodological designs. Convergence across methods increases confidence in the consistency of findings. Third, pilot testing will be used in the experimental phase to strengthen experimental realism and improve generalisability, consistent with best-practice recommendations for organisational experimentation [11]. A pilot sample of 20–30 managers will be used to identify ambiguities and refine materials before the main study. Finally, robustness testing will be conducted across all empirical phases. Longitudinal models will be assessed using alternative specifications, multilevel models will be assessed using alternative nesting structures, and experimental analyses will be tested using alternative applicant profiles to assess consistency.

## Limitations

This framework integrates multiple datasets and methodological designs, which may introduce comparability challenges across data sources and institutional contexts. Longitudinal panel measures may not fully capture neurodivergence-specific workplace experiences, particularly where disclosure is absent or incomplete.

Linked employer-employee datasets may vary in the scope and precision of HR system measurement, potentially limiting comparability across organisations. Experimental simulations necessarily simplify real-world recruitment processes and may not fully capture the complexity of hiring decisions. Accordingly, the framework should be interpreted as a triangulated methodological approach rather than a single unified model. In addition, several variables across the proposed framework rely on self-reported survey responses and are therefore susceptible to recall bias, social desirability bias, inaccurate reporting, and response misclassification. Employees may also choose not to disclose health conditions or disabilities, potentially resulting in measurement error and underestimation of group differences. Although the integration of conceptual synthesis, longitudinal analysis, multilevel modelling, and experimental methods reduces reliance on any single source of evidence, these sources of bias remain inherent limitations of organisational research using self-reported data and should be considered when interpreting the findings.

## Ethics statements

Phase 1 uses publicly available peer-reviewed literature and does not require ethical approval.

Phase 2 uses secondary de-identified panel data from HILDA and is exempt from human ethics approval under institutional guidelines.

Phase 3 uses secondary de-identified linked employer-employee data from WERS and is exempt from human ethics approval under institutional guidelines.

Phase 4 involves human participants and will require institutional ethics approval prior to data collection. All participants will provide informed consent before participation, and participation will be voluntary and anonymous*.*

## Related research article

Tootell, B., & Rao, V. (under review). Defining Neurodiversity for Human Resource Management Research: Integrating Clinical, Educational, and Organisational Perspectives. Submitted to Human Resource Management Review.

## Supplementary material *and/or* additional information [OPTIONAL]

Supplementary materials include variable operationalisation tables for HILDA and WERS, experimental vignette materials, manipulation check measures, and the structured coding template used in Phase 1. These materials are provided to support replication and adaptation across organisational contexts.

## Declaration of competing interest

The authors declare that they have no known competing financial interests or personal relationships that could have appeared to influence the work reported in this paper.

## Figures and Tables

**Table:** 

Construct	Operational definition	Example HILDA indicator	Coding approach
Job demands	Perceived intensity and pressure of work	Workload pressure; time pressure	Standardised composite index
Emotional strain	Emotional burden arising from work	Stress at work	Standardised scale
Job autonomy	Degree of control over work tasks	Freedom in how work is done	Standardised scale
Supervisor support	Perceived managerial support	Supervisor assistance/support	Standardised scale
Job flexibility	Perceived flexibility in work arrangements	Flexibility in start/finish times	Standardised scale
Wellbeing	General psychological wellbeing	Mental health/wellbeing scale	Continuous
Job satisfaction	Overall job satisfaction	Satisfaction with current job	Continuous
Retention	Continued employment across waves	Employment continuity	Binary/longitudinal
Wages	Earnings from employment	Weekly earnings	Logged continuous
Disability status	Self-reported health/disability condition	Long-term health condition	Binary moderator

**Table:** 

Level	Construct	Operationalisation
Organisation	Recruitment structure	Formal interview guides, structured scoring
Organisation	Flexible work practices	Flexible scheduling, remote work policies
Organisation	Supervisor training	Formal manager capability development
Organisation	Performance systems	Formal appraisal systems
Organisation	Employee support	Assistance programmes, support mechanisms
Employee	Job demands	Workload, pressure, interruptions
Employee	Job resources	Support, autonomy, flexibility
Employee	Job satisfaction	Satisfaction rating
Employee	Wellbeing	Self-reported workplace wellbeing

**Table:** 

Study ID	Recruitment structure	Job design	Supervision	Performance management	Work environment
Study 1	Structured/ unstructured	High/low autonomy	High/low support	Explicit/implicit criteria	High/low sensory demand
Study 2	Structured/ unstructured	Predictable/ unpredictable	High/low clarity	Formal/informal	High/low interruption

## Data Availability

Data will be made available on request.

## Appendix A: This appendix outlines the operationalisation of key constructs used in Phase 2 of the multi-method framework using the Household, Income and Labour Dynamics in Australia Survey (HILDA)

Variable construction procedure

All predictor variables will be standardised prior to analysis and combined into demand and resource indices using mean composite scoring. Internal consistency will be assessed using Cronbach's alpha.

## Appendix B: WERS variable operationalisation table This appendix outlines variable operationalisation in Phase 3 using the Workplace Employment Relations Study (WERS)

Aggregation procedure

Organisation-level HR variables are derived from management survey responses. Aggregation adequacy will be assessed using:

• ICC(1)
• ICC(2)
• rwg

Thresholds:

• ICC(1) > .05
• ICC(2) > .60
• rwg > .70

## Appendix C: This appendix contains experimental applicant vignette materials

Experimental vignette template

• Applicant profile (constant across conditions)
• Applicant Name: Jordan Taylor
• Education: Bachelor’s degree in Business Administration
• Experience: Five years in project coordination and process improvement
• Technical capability: High
• Work sample score: High

Manipulation 1: Structured interview

• Interviewer asks all candidates identical job-relevant questions.
• Standardised scoring criteria provided.
• Work sample evaluated independently.

Manipulation 2: Unstructured interview

• Interviewer uses open conversational format.
• No structured scoring criteria.
• Work sample discussed informally.

Manipulation 3: Polished communication style

• Candidate responds fluently, maintains eye contact, provides socially conventional responses.
• Manipulation 4: Less polished communication style
• Candidate responds accurately but with less social fluency, longer pauses, reduced eye contact, and more literal responses.

Outcome measures

• Participants rate:
• Hiring likelihood
• Perceived competence
• Perceived fit
• Decision confidence

Scale

• 1–7 Likert

## Appendix D: This appendix contains manipulation check measures for the experimental vignette phase

To assess the effectiveness of the experimental manipulations, participants will complete a brief set of manipulation check measures immediately following the candidate evaluation task. These measures are designed to confirm whether participants perceived the intended differences in recruitment structure and candidate communication style across the experimental conditions.

Perceptions of recruitment structure will be assessed using two items measuring the extent to which the recruitment process was experienced as formalised and systematically organised. Participants will indicate their agreement with statements such as *“This recruitment process was highly structured”* and *“The candidate was assessed using clear evaluation criteria”* using a seven-point Likert scale ranging from 1 (*strongly disagree*) to 7 (*strongly agree*).

Perceptions of candidate communication fluency will also be assessed using two items designed to capture the extent to which the candidate’s communication style was perceived as polished and socially fluent. Participants will rate their agreement with statements including *“The candidate communicated in a polished and socially fluent way”* and *“The candidate demonstrated strong interpersonal communication”* using the same seven-point response format.

To assess ecological validity, participants will complete a realism check designed to evaluate whether the recruitment scenario was perceived as credible and representative of real-world hiring situations. This will be measured using the item *“This recruitment scenario felt realistic”* on the same seven-point Likert scale.

Responses to these manipulation check items will be analysed prior to hypothesis testing to confirm the integrity of the experimental manipulations. Where manipulation checks indicate insufficient differentiation between conditions, experimental materials will be reviewed and refined prior to full implementation.

## Appendix E: Phase 1 Coding template Each included study should be coded using the following framework

The coding framework is structured around five core organisational domains relevant to neuroinclusive work systems. Recruitment structure refers to the degree of formalisation embedded within hiring processes, including the use of structured interviews, standardised assessments, and explicit evaluation criteria. Job design captures the extent to which work roles provide autonomy, flexibility, and predictability in task completion and workflow management. Supervision refers to the quality and consistency of managerial support, including the provision of feedback, role clarity, and ongoing guidance. Performance management captures the transparency of performance criteria and the extent to which behavioural expectations are explicitly communicated and consistently applied. Finally, work environment refers to the broader contextual features of the workplace, including sensory demands, the frequency of interruptions, and the communication norms that shape day-to-day employee interactions.

Together, these five domains provide a structured framework for identifying how organisational systems shape the accessibility of work environments and influence the demand-resource architecture experienced by employees.
